# Emotional dynamics during glucocorticoid treatment in chronic cluster headache – Insights from dynamic time warp analysis

**DOI:** 10.1016/j.cpnec.2026.100385

**Published:** 2026-10-02

**Authors:** Paulien J. van Tilborg, Anne-Sophie C.A.M. Koning, Willemijn C. Naber, Onno C. Meijer, Rolf Fronczek, Erik J. Giltay

**Affiliations:** a Department of Neurology, Leiden University Medical Center, Leiden, the Netherlands; b Department of Sleep and Cognition, Netherlands Institute for Neuroscience, Amsterdam, the Netherlands; c Department of Psychiatry, Amsterdam UMC, Amsterdam, the Netherlands; d Department of Endocrinology, Leiden University Medical Center, Leiden, the Netherlands; e Stichting Epilepsie Instellingen Nederland (SEIN), Sleep-Wake Centre, Heemstede, the Netherlands; f Department of Psychiatry, Leiden University Medical Center, Leiden, the Netherlands; g Collaborative Antwerp Psychiatric Research Institute (CAPRI), Department of Biomedical Sciences, University of Antwerp, Belgium

**Keywords:** ‘Cluster headache’, ‘Dynamic time warping’, ‘Glucocorticoids’, ‘Prednisone’, ‘Network analysis’

## Abstract

**Background:**

Cluster headache can affect mental health due to the severe attacks, with high rates of depression and anxiety. When prophylactic treatment fails, high dose prednisone can be used as bridging therapy, but may cause neuropsychological side effects. This prospective, exploratory, observational pilot study, aimed to assess the temporal associations between emotions during glucocorticoid treatment in people with cluster headache.

**Methods:**

During prednisone treatment (67 days), ten participants with chronic cluster headache completed daily questionnaires consisting of ecological momentary assessments regarding emotion, cluster headache bother and attack frequency. Undirected and directed dynamic time warp (DTW) analyses were used to examine the temporal relationship between symptoms at individual-level and group-level.

**Results:**

The GAMM modelled levels of individual emotions showed a monotonic increase of negative emotions and decrease of positive emotions during follow-up. Individual-level DTW analyses showed high interindividual variability. Group-level DTW analyses found five symptom clusters with similar dynamics. Feelings of upset, hopelessness and low motivation showed the highest outstrength (temporal-lead) and tended to precede changes in other emotions. Cluster headache bother and attack frequency were not interconnected. Changes in attack frequency had temporal associations with subsequent emotional changes, whereas changes in cluster headache bother tended to be preceded by emotional changes, although both cluster headache items demonstrated a limited number of connections in the network plots.

**Conclusion:**

At the end of glucocorticoid treatment, participants experienced less positive and more negative emotions. Temporal associations were mainly present between emotions. Cluster headache bother was associated with preceding adverse emotions rather than attack frequency, emphasizing the importance to include cluster headache bother when assessing the efficacy of prophylactic treatment. As our exploratory results showed temporal lead for feeling upset, low motivation and low hopefulness, tracking such symptoms may facilitate personally tailored interventions during a glucocorticoid treatment course.

## Graphical abstract

**Figure:**
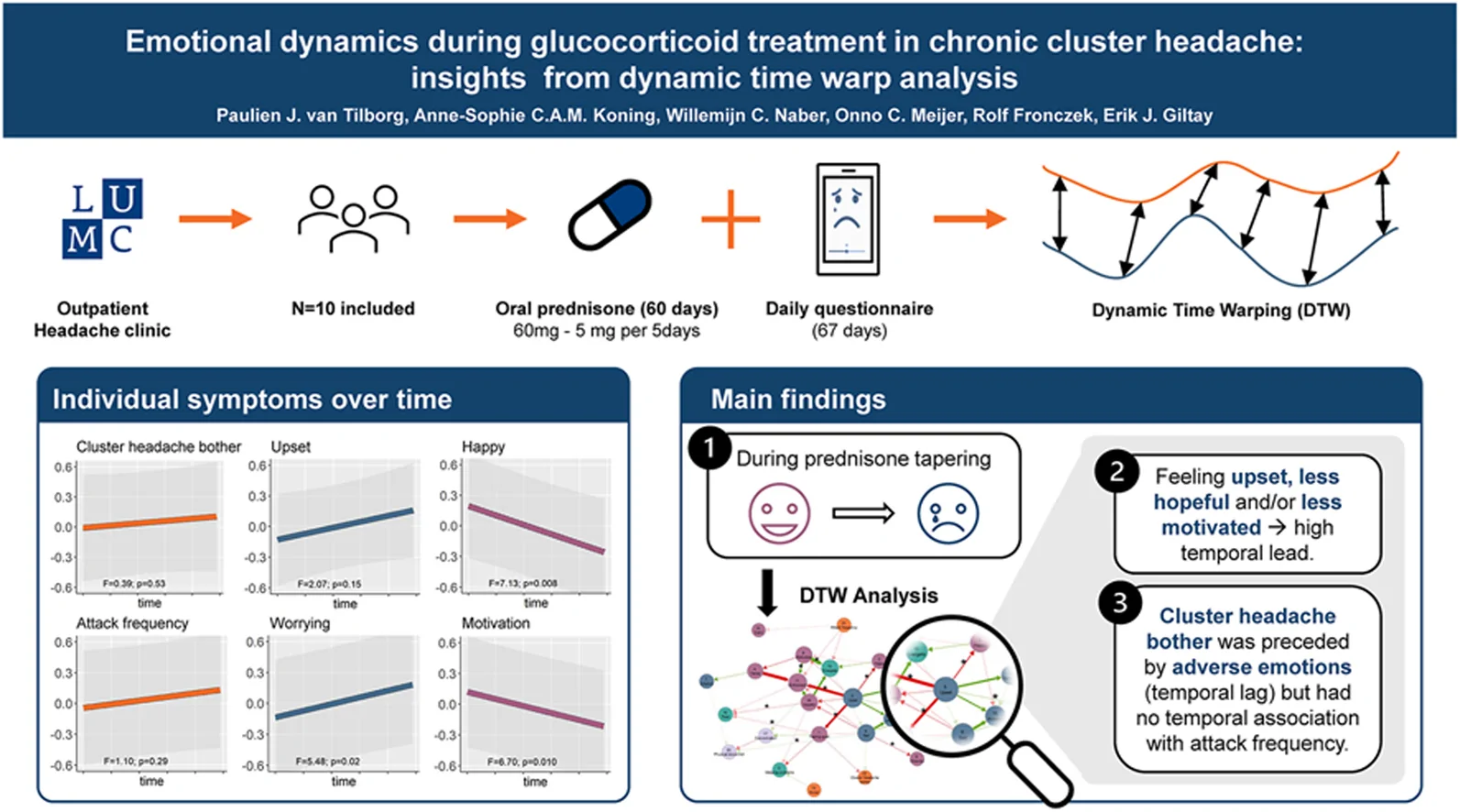

## 1 Introduction

Cluster headache is a trigeminal autonomic cephalalgia characterized by excruciating unilateral headache attacks up to eight times per day, accompanied by restlessness and/or ipsilateral autonomic symptoms [1]. While episodic cluster headache with attack free intervals is most common, about 15% experiences chronic cluster headache [1]. It is considered highly disabling and greatly impacts quality of life [[2], [3], [4]]. The odds of depression are nearly three times higher in chronic cluster headache compared to controls, with up to 60% experiencing depression during their lifetime [5]. People with chronic cluster headache also report increased levels of anxiety and hopelessness, and mood related symptoms tend to be more severe in chronic cluster headache compared to episodic cluster headache [5,6]. Psychological factors, such as fear of attacks, depression and anxiety are stronger predictors for pain-related disability than attack frequency [4,7].

Given the frequent attacks, the need for prophylactic treatment is high in chronic cluster headache. Unfortunately, commonly prescribed prophylactics such as verapamil, topiramate and lithium and more invasive treatments like neuromodulation are sometimes insufficient. In such cases, oral glucocorticoids can sometimes temporarily reduce attack frequency [8]. Due to the risk of severe side effects, chronic glucocorticoid use is considered unsafe and it should only be used as bridging or additive therapy in severe chronic cluster headache [8], or as short-term preventive therapy in episodic cluster headache [9]. However, even short-term glucocorticoid use can lead to neuropsychological side effects [10]. These side effects usually present as mood changes such as irritability or anxiety, cognitive or behavioral changes such as hyperactivity, or sleep disturbances, although they can be more severe and result in mania, depression or even suicidality in some cases [11].

Despite the high rate of psychological symptoms related to cluster headache, studies examining the efficacy and safety of glucocorticoids in cluster headache did not include a comprehensive evaluation of potential mood-related side effects, with a generalized depression scale being used at most [9]. Therefore, we aimed to assess the temporal associations between emotions during glucocorticoid treatment in people with cluster headache, using a symptom network approach. Dynamic Time Warp (DTW) analyses were performed to assess the temporal dynamics at individual- and group-level. This approach allowed us to examine temporal associations between changes in specific emotions and overall emotional well-being, which may help identify emotional changes that warrant closer monitoring during glucocorticoid treatment. We anticipated that the individual network plots would reveal interindividual variability, while the group-level plots might highlight several emotions with strong temporal associations to other emotions and potentially highlight emotions associated with changing cluster headache symptoms.

## 2 Method

### 2.1 Design and study population

Participants for this prospective, exploratory, observational pilot study (ClinicalTrials.gov; ID: NCT05391295) were recruited via the outpatient headache clinic at the Leiden University Medical Center in the Netherlands. Participants completed a daily questionnaire during glucocorticoid treatment to monitor emotions, cluster headache attack frequency and how much they were bothered by their cluster headache. Patients were eligible if they were i) diagnosed with chronic cluster headache according to the International Classification of Headache Disorders 3rd edition criteria, ii) prescribed a course of oral prednisone for cluster headache, iii) ≥16 years of age and iv) had sufficient proficiency in Dutch to complete the questionnaires. No specific exclusion criteria were defined. Inclusions continued until the intended sample size of ten was reached. The Institutional Review Board determined that the study did not fall under the scope of the Medical Research Involving Human Subjects Act (in Dutch: Wet Medisch-wetenschappelijk Onderzoek met mensen) and was exempted for additional medical ethical review, as the questionnaire was deemed non-burdensome and implemented no other behavioral protocols (W2022.002). Written informed consent prior to inclusion was sufficient. Data was collected between 29 March 2023 and 28 July 2024.

### 2.2 Procedures and EMA questionnaire

Participants were included at the start of glucocorticoid treatment. In our center, prednisone prescription for cluster headache follows a standard protocol with a starting dose of 60 mg/day, tapered by 5 mg every 5 days. Participant monitoring using daily questionnaires continued until 7 days after the final dose, resulting in a 67-day follow-up period. Questionnaires were completed in the form of ecological momentary assessments (EMA), using the m-Path app on a personal smartphone or tablet. EMA can facilitate the collection of time-series data in real-world settings [12,13]. Individuals repeatedly answer the same questions at a set interval over an extended period, allowing for dynamic symptom monitoring with limited risk of recall bias. It is suitable for the assessment of fluctuating experiences like emotions and disease symptoms. M-path (www.m-Path.io) is an online platform that enables EMA by sending questionnaires at a predefined schedule [14]. Every day at 8.00 p.m., the m-Path app sent a push notification indicating the questionnaire was available. All questions had to be answered in relation to the preceding 24 h. To maximize compliance, participants were stimulated to complete the questionnaire immediately upon receiving the alert. A reminder was sent if the questionnaire was not completed by 9.00 p.m. At midnight, the questionnaire was automatically locked to ensure all data entries were made on the same day the data corresponded to.

The EMA questionnaire (Appendix A) consisted of 23 short questions, including 21 general mood and physical well-being items and 2 cluster headache specific items (*cluster headache bother* and *attack frequency*). A continuous slider ranging from 0 (not at all) to 100 (very) was used to answer all questions, except for *attack frequency*, which was a multiple choice question consisting of the options *no attacks*, *1 attack*, *2-4 attacks*, *5-10 attacks* and *>10 attacks*. For some mood items, the slider was supported by a smiley face, which's expression changed based on the slider position (e.g. sad to happy expression).

### 2.3 Statistical analysis

#### 2.3.1 Dynamics of individual items during follow-up

To describe the change of individual items during prednisone use, generalized additive mixed models (GAMMs) were fitted, including a participant-specific random intercept and a first-order autoregressive residual correlation structure to account for temporal dependency between consecutive daily assessments. The nonlinear effect of time was retained as a smooth term.

#### 2.3.2 Dynamic time warp analysis

DTW analysis was used to analyze item dynamics, resulting in network plots [15]. Data were group-level standardized before DTW analysis. The network plots served as a proof-of-principle visualization of the overall pattern of temporal relationships between symptoms. They summarize the relationships between items by demonstrating the (i) existence, (ii) strength and (iii) statistical significance of connections between questionnaire items. A dense network plot features many interconnected items, while network plots can also be more sparsely connected. DTW is a non-linear clustering technique that aligns the standardized scores in time-series data. The algorithm uses a cost matrix to calculate the minimally warped path (the lowest cumulative distance) between time points of two data sequences. Starting in the lower left corner of the matrix, a path to the upper right corner is found by taking steps with the lowest increase in cost distance, resulting in a total distance score [16]. When items follow a similar pattern over time, the distance between these items will be small; whereas if items follow a different pattern, the distance will be large. More detailed information on DTW analysis was published previously [17]. Other studies utilizing DTW demonstrated that item dynamics over time are appropriately portrayed in network plots [[17], [18], [19], [20], [21], [22], [23]].

DTW was applied to the EMA data at two levels: within participants (individual-level) and between participants (group-level*).* At both levels, *undirected* and *directed* DTW analyses were performed. Undirected analysis identifies covariation between items over time, regardless of whether changes occur slightly earlier for A or B, and thus captures broadly similar trajectories, regardless of which one precedes the other. Directed analysis incorporates temporal ordering, identifying instances in which changes in one item tend to precede changes in another item within the specified time window (i.e. changes in A are followed by changes in B). These lead-lag associations indicate temporal sequence but should not be interpreted as evidence of causal influence. For the directed group-level analysis, the instrength (temporal lag) and outstrength (temporal lead) of all items were determined. These are measures of (i) the number and (ii) strength of in- and outcoming arrows for each individual node and thus summarize its position within the complete pattern of directed associations rather than testing a single edge. The overall in- and outstrength of each item served as the main interferential analysis and was presented in a forest plot, adjusted for the available confounders: age and sex. In- and outstrength estimates were corrected for multiple testing using the Benjamini-Hochberg false discovery rate procedure. High outstrength indicates that changes in an item tend to precede changes in multiple other items within the network, whereas high instrength indicates that changes in an item tend to be preceded by changes in other items. As DTW relies on fluctuating variables to identify temporal relationships and thus cannot identify connections between emotions and stable cluster headache items (i.e. full remission or no response to treatment), sensitivity analyses based on treatment response were performed. Additionally, sensitivity analyses were performed to assess whether the observed associations were influenced by comorbidities that may affect emotions.

#### 2.3.3 Undirected analyses algorithm

First, individual undirected distance matrices were generated and visualized in a separate network plot per participant. Subsequently, results were aggregated at the group-level, by comparing the mean distance between each symptom pair with all other symptom distances using t-tests. If the mean distance between a symptom pair was smaller than the other distances, this similarity in symptom dynamics was considered stable. Statistically significant (p < 0.05) edges were indicated by an asterisk. Items were clustered in groups based on similarity, using a hierarchal cluster analysis with the ‘Ward.D2’ clustering method. The step pattern ‘symmetric2’ and the global constraint ‘Sakoe-Chiba’ with a window size of 1 were applied. This ensured that only changes between one time-point before or one time-point after a datapoint were used for warping [15]. Because DTW has no flexibility to warp observations before the beginning or beyond the end of a time series, differences between the first or last observations of two trajectories may contribute disproportionately to the total distance. To reduce this boundary effect, we used linear interpolation to create a finer temporal grid between consecutive assessments. These five interpolated values were not considered additional independent observations. The DTW window was increased proportionally with the interpolation factor, such that the maximum allowable temporal displacement remained equivalent to one original daily assessment. As a sensitivity analysis, we repeated the analyses using ten rather than five interpolated values to assess whether results depended on the chosen interpolation density.

#### 2.3.4 Directed analyses algorithm

The directed analyses largely used the same DTW algorithm and statistical tests as described above, with one major adjustment. For the directed distance matrices, an adapted version of the ‘Sakoe-Chiba’ band was used which set the window type to asymmetric. This ensured that symptoms could only be aligned in a forward direction, following the natural progression of time. The algorithm calculated the relative difference in alignment-based distance between two symptoms in both directions, i.e. A to B and B to A. This value ranged from −1 to +1, with +1 indicating that changes in A were followed exactly by changes in B one timepoint later. The final distance is based on the relative difference between these two values, with higher positive scores indicating a stronger temporal influence of one symptom on the other. We tested whether the difference between each pair of nodes differed significantly from zero using t-tests at group-level. If the mean directed distance between a symptom pair differed from zero with p < 0.15, this temporal relationship was indicated by an arrow, and significant (p < 0.05) arrows were indicated by an asterisk. The p < 0.15 threshold was chosen as an exploratory display threshold to obtain an interpretable network, and to show the edges on which the in- and outstrength values were based.

#### 2.3.5 General statistics

For this exploratory pilot study, a sample size of ten was chosen. Participant characteristics were described by means (SD or range) and medians (inter quartile range; IQR). All statistical analyses were performed using RStudio statistical software (R version 4.2.3; R Foundation for Statistical Computing, https://www.R-project.org/). The packages ‘‘parallelDist’’ (version 0.2.6), ‘‘mgcv’’ (version 1.9.1), ‘‘dtw’’ (version 1.23.1), ‘‘qgraph’’ (version 1.9.8), ‘‘tidyverse’’ (version 2.0.0) and ‘‘lme4’’ (version 1.1.35.5) were installed. P-values smaller than 0.05 were considered statistically significant.

#### 2.3.6 Data availability

The data that was analyzed during the present study is available from the corresponding author on reasonable request.

## 3 Results

### 3.1 Participant characteristics

The Participants (n = 10) had a mean age of 44.8 (SD 13.8) and 6 (60%) were female (Table 1). In 9 participants (90%) prednisone was prescribed per standard protocol. In 1 participant (10%), the dose was reduced every 7 days instead of 5 to prolong the beneficial effect, which they previously only experienced at higher doses. This resulted in a mean follow-up of 62.7 complete days of assessments (range 53 – 92). Mean questionnaire compliance was 88.8% (range 78% – 100%). Median daily attack frequency before prednisone was 7 [IQR: 4-9]. During prednisone use, 30% of participants became attack-free for the majority of the follow-up period (responders: IDs 6, 8 and 9), 40% experienced less cluster headache attacks (partial responders: IDs 2, 4, 5 and 10), and 30% had no clinically relevant response (non-responders: IDs 1, 3 and 7; Appendix B).

**Table 1:** Participant characteristics.

	Total (N = 10)
Age, years	44.8 ± 13.8
Chronic CH	10 (100%)
Sex	
*Male*	4 (40%)
*Female*	6 (60%)
Baseline Attack frequency*	7 (4-9)
Daily prednisone dose	
60 mg*, tapered with*5 mg per *5 days*	9 (90%)
60 mg*, tapered with*5 mg per *7 days*	1 (10%)
Complete days of assessments, mean (range)	63 (53-92)
Relevant comorbidities	
*Unspecified bipolar disorder*	*1 (10%)*
*Paroxysmal Hemicrania*	*1 (10%)*

All variables are depicted as mean ± SD (standard deviation), median (IQR; interquartile range) or number (%) unless otherwise specified. *Daily attack frequency before start of glucocorticoid treatment.

### 3.2 Dynamics of individual items during follow-up

The GAMM modelled levels of all individual items over time are shown in Fig. 1. Most items showed a monotonic trajectory over time, with positive emotions (*feeling good*, *happy*, *cheerful*, *motivation*, *enthusiasm*, *calm*, *hopeful*) decreasing and negative emotions (*upset*, *irritation*, *sad*, *nervous*, *anxious*, *guilty*, *worrying*, *tense*) increasing. Overall, half of the items, including many positive emotions, showed a statistically significant change over time. For example, *feeling good* and *happy* decreased (F = 14.01, p < 0.001 and F = 7.13, p = 0.008), whereas *upset* and *irritation* increased (F = 2.07, p = 0.15 and F = 5.34, p = 0.02). Both *attack frequency* and *cluster headache bother* followed a slight upward monotonic trajectory, but did not show a statistically significant change (F = 1.10, p = 0.29 and F = 0.39, p = 0.53). Sleeping problems decreased during follow-up, whereas other physical symptoms (such as *energetic, tired)* worsened. Physical discomfort was the only non-monotonic item and followed an inverted U-shape. Sensitivity analyses showed similar trajectories of the individual item plots of responders, partial responders and non-responders respectively, except for both cluster headache items.

**Fig. 1:**
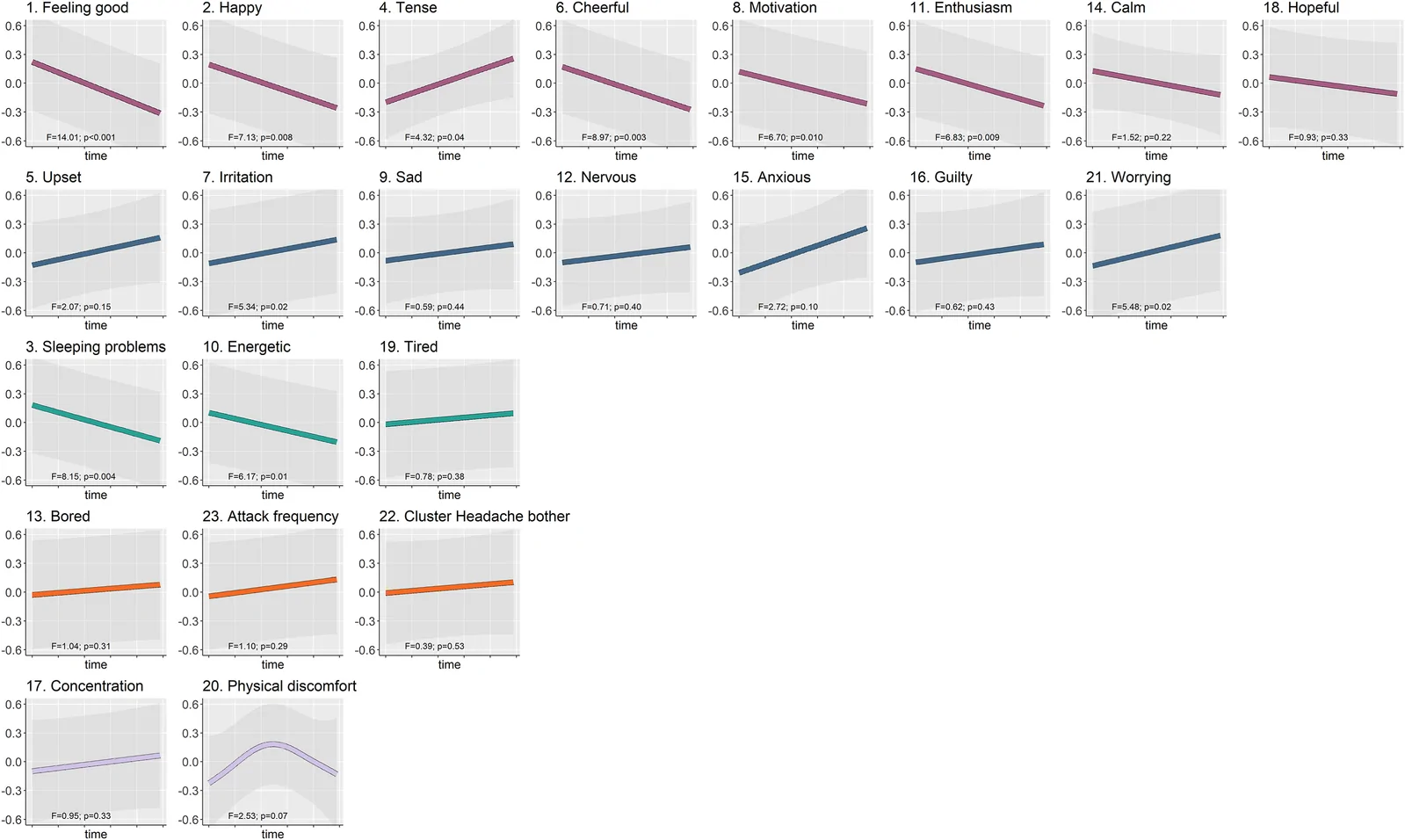
Individual items over time. All individual GAMM-modelled symptoms over time (x-axis). The items are separated into five different symptom groups, each indicated by a different color. The coloring and grouping of variables are based on the group-level undirected analysis presented in Fig. 3A.

### 3.3 Individual-level DTW analyses

Individual-level undirected and directed DTW analyses resulted in network plots for all participants (Appendix B). In 3 (30%) of the directed individual-level network plots, the cluster headache items were connected to emotion items (two responders, one partial responder). Since large interindividual variations were observed, two illustrative examples are highlighted: one case with and one case without clinical effect of prednisone on their cluster headache (Fig. 2). In participant 3, *cluster headache bother* temporarily decreased at the start of treatment whilst *attack frequency* remained stable during the prednisone course. DTW resulted in a high-density network plot with multiple connections within and between item groups. Most notably, increases in *cheerful* and motivation were temporally associated with a subsequent decline of *cluster headache bother*, whilst an increase of *attack frequency*, *anxious* and *worrying* were temporally associated with a subsequent increase of *cluster headache bother*. Participant 6 was completely attack-free during prednisone treatment. Consistent with the absence of fluctuations in *attack frequency* and *cluster headache bother,* DTW analysis resulted in minimal to no connections with other items in the network, as it relies on identifying temporal relationships between fluctuating variables.

**Fig. 2:**
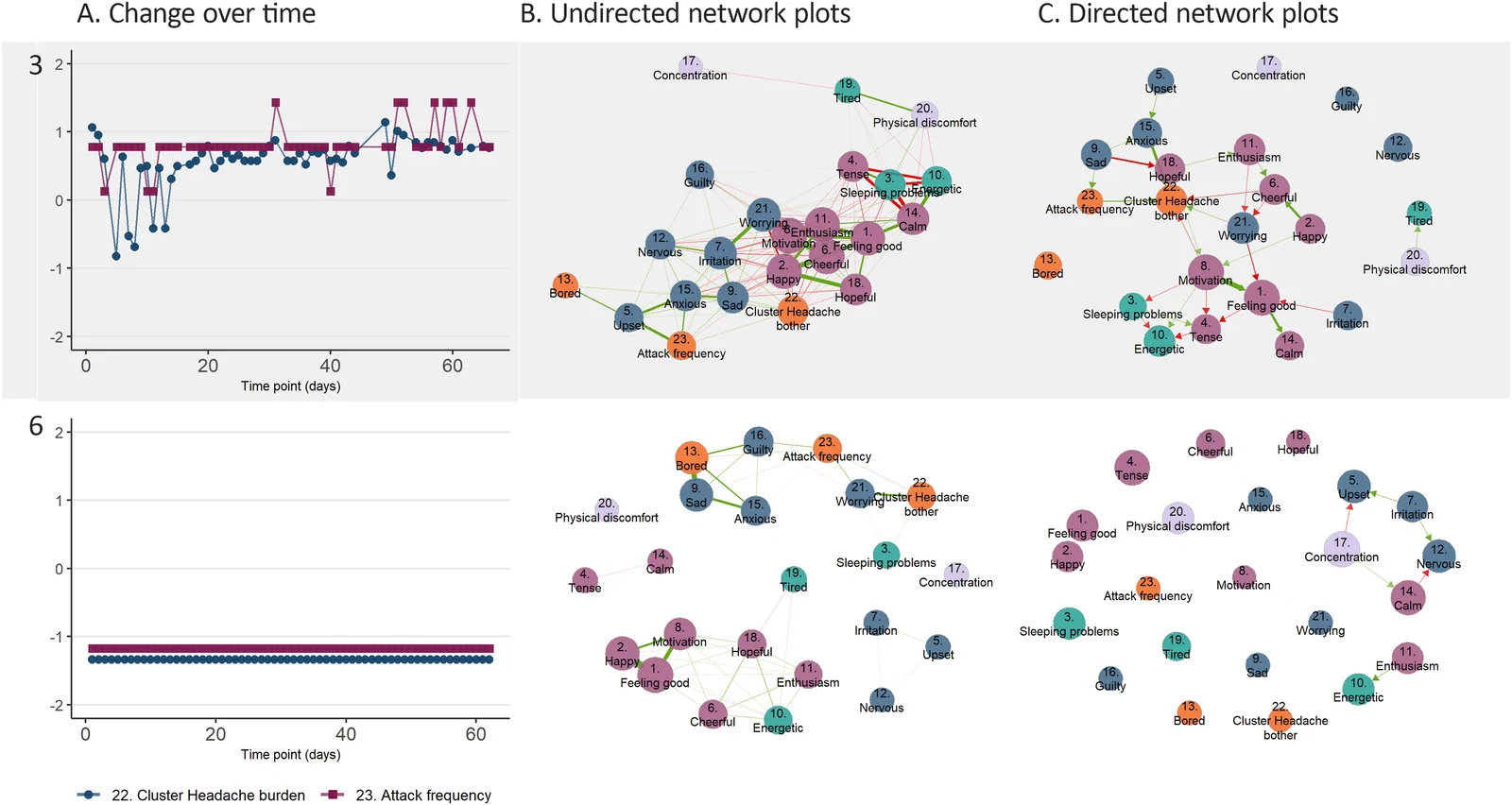
Individual-level dynamic time warp analysis of two participants. A: Cluster headache symptoms; portrays standardized scores of cluster headache bother and attack frequency. A high score indicates no treatment response, and a low score indicates good treatment response. B: undirected network and C: directed network. The size of each node is proportional to its connectivity. The darkness and thickness of the lines (undirected) and arrows (directed) between the nodes correspond to the strength of the effect, with darker colors and bolder lines/arrows indicating stronger effects. Green and red lines/arrows represent positive and negative effects respectively. A negative value, represented by a red edge or arrow, indicates that changes in A co-occur with opposite changes in B. The coloring of variables are based on the group-level undirected analysis presented in Fig. 3A. Data is shown for two illustrative individuals: one case without (ID 3; top) and one case with (ID 6; bottom) clinical effect of prednisone on cluster headache. Individual plots for all ten participants can be found in Appendix B.

### 3.4 Group-level DTW analyses

#### 3.4.1 Undirected analysis

A hierarchal cluster analysis identified five item groups that followed similar paths in the undirected group-level analysis (Fig. 3A). Cluster 1 (turquoise) consisted of *tired*, *energetic*, and *sleeping problems*. Cluster 2 (lilac) consisted of *physical discomfort* and *concentration*. Cluster 3 (orange) included *attack frequency*, *cluster headache bother*, and *bored*. Cluster 4 (blue) incorporated the negative emotions, namely *upset*, *nervous*, *irritation*, *worrying*, *sad*, *anxious*, and *guilty* and cluster 5 (pink) incorporated mostly positive emotions and consisted of *happy*, *feeling good*, *cheerful*, *motivation*, *hopeful*, *enthusiasm*, *tense*, and *calm*. The undirected network analysis mainly yielded connections within the symptom clusters (Fig. 3B). The positive emotion cluster (pink/cluster 5) showed the most temporal associations, with 18 significant (p < 0.05) associations within the group. *Feeling good*, *happy*, *motivation*, *cheerful* and *enthusiasm* had strong positive temporal associations with each other, and negative temporal associations to *tense* and *tired*. Within the negative emotion cluster (blue/cluster 4), there were 4 significant (p < 0.05) temporal associations, with the strongest association between *sad* and *anxious*. The cluster headache items (orange/cluster 3) were connected to the negative emotion cluster via *sad*. *Upset* was the bridging node that connected the negative and positive emotion cluster.

**Fig. 3:**
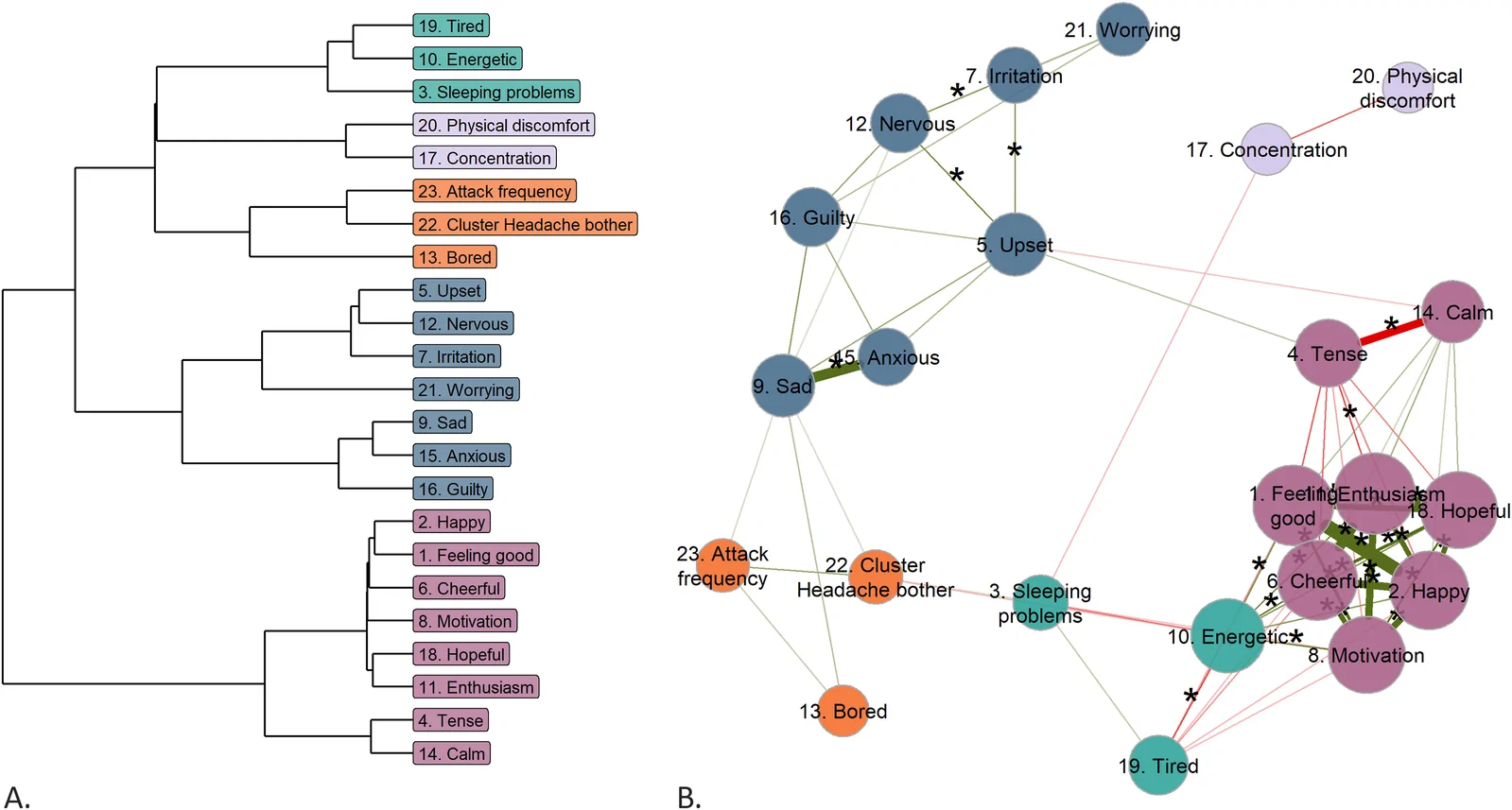
Undirected group-level dynamic time warp analysis. A: dendrogram of the clustered emotions, resulting in 5 symptom groups. B: undirected network plot, identifies associations between items regardless of their temporal order (A and B are connected, regardless of direction). The size of each node is proportional to its connectivity. The darkness and thickness of the lines between the nodes correspond to the strength of the effect, with darker colors and bolder lines indicating stronger effects. Green and red lines represent positive and negative effects respectively. A negative value, represented by a red edge or arrow, indicates that changes in A co-occur with opposite changes in B. All connections with p < 0.15 are shown in the network plot (p < 0.15 threshold was chosen as an exploratory display threshold to obtain an interpretable network). An asterisk (*) indicates p < 0.05.

#### 3.4.2 Directed analysis

Fig. 4A demonstrates the results of the directed group-level network analysis. The instrength and outstrength of each item were calculated to quantify their temporal association (Fig. 4B). Several items showed high in- or outstrength, although most did not reach statistical significance after correction for multiple testing. Nevertheless, some items showed potentially relevant temporal associations. *Upset* had the highest outstrength, implying that changes in *upset* tended to be followed by similar changes in *anxious* and *nervous* and opposite changes in *happy*, *enthusiasm* and *feeling good* the next day. Other items with high outstrength were *motivation* and *hopeful*. Changes in *motivation* or *hopeful* tended to be followed by similar changes in *energetic* and *enthusiasm* and inverse changes in *tense* and *tired* respectively. The highest instrength was found for *energetic, cheerful* and *physical discomfort,* implying a temporal association with changes in other items the previous day. Changes in energetic tended to be preceded by changes in *motivation*, *happy* and *hopeful,* whereas changes in *physical discomfort* were preceded by changes in *irritation*, *sad* and *concentration*. Sensitivity analyses based on the comorbities listed in Table 1 did not change the results of the network analysis.

**Fig. 4:**
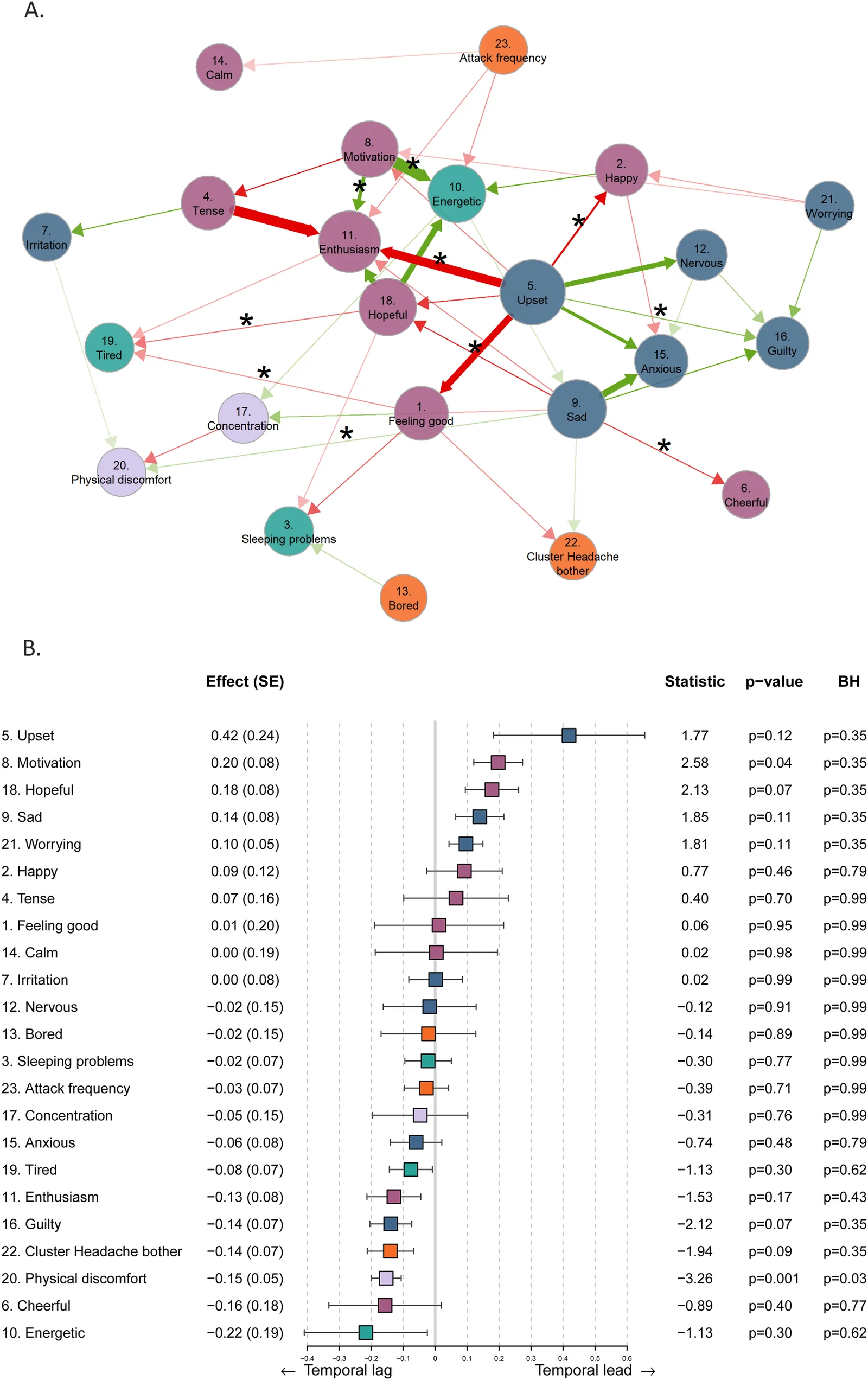
Directed group-level analysis. Panel A (top): directed group-level network plot: identifies temporal associations between items (i.e. A is followed by B the next day). These lead-lag associations indicate temporal sequence but should not be interpreted as evidence of causal influence. The size of each node is proportional to its connectivity. The darkness and thickness of the lines between the nodes correspond to the strength of the effect, with darker colors and bolder lines indicating stronger effects. Green and red lines represent positive and negative effects respectively. A negative value, represented by a red edge or arrow, indicates that changes in A co-occur with opposite changes in B. All connections with P < 0.15 are shown in the network plot (p < 0.15 threshold was chosen as an exploratory display threshold to obtain an interpretable network). An asterisk (*) indicates P < 0.05.Panel B (bottom): Forest plot, portraying the instrength (temporal lag) and outstrength (temporal lead) of each item, which are measures for (i) the number and (ii) strength of in- and outcoming arrows for each individual node. High outstrength indicates changes in that item will be followed by changes in other items. High instrength indicates changes is that item were preceded by changes in other items. The plot is adjusted for age and sex. In- and outstrength estimates were corrected for multiple testing using the Benjamini-Hochberg (BH) false discovery rate procedure.

#### 3.4.3 Cluster headache items in directed group-level analysis

The two cluster headache items were not connected to each other in the directed DTW analysis, although the network plot did show some suggestive temporal associations between the respective cluster headache items and other emotions. *Attack frequency* was inversely associated with *calm*, *enthusiasm* and *energetic*, suggesting that changes in these items tended to follow changes in attack frequency the previous day. The network plot showed no items that preceded changes in *attack frequency*. *Cluster headache bother* showed a suggestive temporal association with sadness and feeling good: changes in sadness and feeling good tended to precede changes in reported *cluster headache bother*. Sensitivity analyses based on treatment response showed no differences, except for *attack frequency* in the partial responder group (who had the highest fluctuation of cluster headache items), in which it was no longer connected to any other item in the directed network plot.

## 4 Discussion

This exploratory pilot study investigated the temporal associations of emotional fluctuations during glucocorticoid treatment in cluster headache. The analyses demonstrated high interindividual variability as expected and identified several potentially relevant temporal associations at the group-level. However, our group-level analysis identified a limited number of temporal relationships between cluster headache and emotion. Moreover, a decrease of positive emotions and increase of negative emotions was observed over time, independent of the treatment effect on cluster headache. Changes in *upset* and *motivation* had the largest outstrength and were associated with of further declines in other emotions the following day. Thus, over time, glucocorticoid treatment was associated with feelings of upset, low motivation, and subsequently other negative emotions, highlighting the importance of informing patients about the potential mood-related side effects.

Emotional fluctuations and particularly deterioration occurred in most participants. Sensitivity analyses showed that this could not be fully explained by treatment response – based on the level of variation within the cluster headache items, on which the model relies to identify temporal associations – as the emotional trajectories were comparable across all responders statuses and did not differ from the main analysis. This suggests that the observed patterns were not simply driven by the effect of prednisone on cluster headache symptoms, and implies that other factors, such as the pharmacological effects of prednisone, treatment expectations and regression toward the mean, are involved. However, we cannot determine the extent to which each of these factors may have contributed to the observed emotional fluctuations. A previous study, partly conducted by our research group (AK, OM, EG), that used DTW to assess emotional dynamics in patients treated with glucocorticoids for itchiness and skin problems resulting from cutaneous T-cell lymphoma, also found that mood and emotion declined at the end of glucocorticoid treatment, with decreased energy, motivation, and enthusiasm [23]. However, contrary to our study, they found an association between increased disease symptoms and low positive emotions the day before. Additionally, our data demonstrated a more pronounced overall decline in mood and emotion. A possible explanation for these differences is the treatment duration, as participants of this study received higher doses for a longer duration compared to the T-cell lymphoma study [23]. Exogenous glucocorticoids have previously been shown to result in adrenal suppression and affect cortisol secretion [24]. Higher doses, frequent (daily) use and treatment duration >14 days all increase the risk of glucocorticoid-induced adrenal insufficiency or glucocorticoid withdrawal syndrome. These risks can be minimized by gradual tapering [24]. As participants in our study used prednisone for ∼60 days, the monotonic temporal emotional changes we observed might thus be partially explained by the cumulative effects of prednisone exposure directly on target tissues or hypocortisolism. Despite prednisone being lowered gradually in our participants, it is plausible that adrenal cortisol secretion was decreased during treatment, although we did not measure this in the present study. This could have contributed to the emotional changes observed in our participants – which persisted during dose tapering – since suppressed adrenal glands may require some time to resume normal hormone production after prednisone is completely ceased.

Although the cluster headache items showed a limited number of temporal associations to individual emotions, some interesting differences emerged between *cluster headache bother* and *attack frequency* in the network plots. Increased *attack frequency* tended to precede a decrease in positive emotions, whereas *cluster headache bother* appeared to follow negative emotional changes the previous day. These temporal associations suggest that emotional fluctuations may influence the perceived bother of cluster headache without changing more objective disease symptoms such as attack frequency, and reflect the complex interaction between emotions and self-reported disease bother. This is further supported by the absence of a temporal relation between the cluster headache items, suggesting that changes in attack frequency are not consistently followed by changes in disease bother 24 h later. This finding is intriguing since the efficacy of prophylactic treatment is typically assessed by the reduction of attack frequency [25]. However, in line with other studies, our data suggest this approach might not fully address the bother patients experienced from their cluster headache.

In previous research, fear of attacks was found to be a significant predictor of cluster headache related disability. Its influence was much stronger than attack frequency, even after adjusting for depression and anxiety [7]. Another study found quality of life was more closely associated with mental health and restrictions in daily functioning than with attack frequency [4]. Given that people with cluster headache are at increased risk of developing internalizing disorders such as depression and anxiety [5,6], additional support along with medication may be necessary to effectively manage cluster headache.

*Upset, motivation* and *hopeful* had the highest outstrength and were associated with subsequent negative changes of other emotions. Their central role in the network was observed in many of the individual network plots, as well as in the group-level analysis. Low mood (i.e. feeling upset, unmotivated or not hopeful) has been described as an important side effect by 43% of patients that used glucocorticoids [26], and compared to controls, people with cluster headache score significantly higher on scales assessing hopelessness [6]. This supports the clinical relevance of the high-outstrength emotions identified by our analysis. In our cohort, dense networks (in which changes in high-outstrength emotions are more likely to trigger domino effects) [27] were mostly found in participants with fluctuating cluster headache symptoms, implying these participants might be more vulnerable for a negative emotional cascade. As a decline in emotional well-being was observed in most participants and temporally associated with a subsequent increase in *cluster headache bother* in some, clinicians should consider mood- and emotion related medical history when prescribing prednisone and inform patients they could temporarily feal more negative, upset or worn-out during treatment. As previous studies have shown DTW analysis can capture high outstrength emotions and their subsequent temporal associations on an individual-level [16,18,19,22], DTW might be developed for application in personalized medicine, although future research should expand on the current evidence to support it's usability. This might be of interest for monitoring during glucocorticoid treatment cluster headache as well. If an individual baseline network is created and identifies emotions with high outstrength prior to glucocorticoid treatment, continuing EMA during treatment may help identify early signs of emotional decline, which could provide clinicians with an opportunity to act on these side-effects.

This prospective pilot study with a long follow-up and daily assessments gives a unique insight into emotional dynamics of people with cluster headache. One of its strengths is the innovative method, using DTW analyses to describe temporal relationships between emotions and cluster headache. The high compliance (88.8%) and its ability to eliminate the risk of recall bias while also minimizing the influence of a particularly good or bad day, confirm the usability of DTW in people with cluster headache. However, some limitations should be discussed. The relatively small sample size of ten limits the precision and generalizability of the group-level network estimates. Additionally, directed DTW analysis identifies temporal lead-lag patterns but cannot establish causality. Findings should thus be considered exploratory, and suggestive temporal associations require replication in larger samples. Another limitation is the absence of baseline data prior to prednisone use, which was not feasible as participants were recruited directly from clinical care and treatment could not be delayed for research purposes. Consequently, we are unable to separate drug-effect from natural disease course, regression towards the mean and treatment expectation, and time-varying confounding cannot be fully excluded. Additionally, although we corrected for age and sex and performed sensitivity analyses based on comorbid diseases that may affect emotion, the magnitude or pattern of emotional changes may differ between clinically relevant subgroups and residual confounding may exist. Finally, an important consideration is that we assessed *cluster headache bother* with a single, relatively unspecific question (Appendix A, item 22). Reponses may therefore encompass different aspects, such as pain severity, anticipatory anxiety, disability, emotional distress or attack frequency. While the specific aspects contributing to bother may vary between individuals, the broad wording allowed participants to capture the aspects most relevant to them at each assessment, and we expect this interpretation to be sufficiently consistent within individuals across repeated assessments to capture changes over time. However, at the group level, *cluster headache bother* may reflect a broad range of patient-relevant experiences.

## 5 Conclusion

We demonstrated that positive emotions seemed to decrease and negative emotions seemed to increase during prednisone treatment in people with cluster headache. Additionally, we found temporal associations between feelings of upset, low motivation and hopelessness and subsequent other emotions in the directed analysis. Cluster headache bother showed temporal associations with emotional fluctuations rather than attack frequency, emphasizing the importance to include patient-reported disease burden when assessing the efficacy of prophylactic medication. Given the small sample size, the group-level findings should be interpreted as exploratory. Larger studies incorporating a baseline period and control group are needed to validate these findings and further develop DTW-based personalized monitoring.

## CRediT authorship contribution statement

**Paulien J. van Tilborg:** Writing – original draft, Project administration, Investigation, Formal analysis, Data curation. **Anne-Sophie C.A.M. Koning:** Writing – review & editing, Project administration, Investigation. **Willemijn C. Naber:** Writing – review & editing, Investigation. **Onno C. Meijer:** Writing – review & editing, Conceptualization. **Rolf Fronczek:** Writing – review & editing, Supervision, Resources, Conceptualization. **Erik J. Giltay:** Writing – review & editing, Visualization, Software, Resources, Formal analysis, Data curation, Conceptualization.

## Declaration of competing interests

The authors declare that they have no known competing financial interests or personal relationships that could have appeared to influence the work reported in this paper.

## Footnotes

Appendix A Supplementary data to this article can be found online at https://doi.org/10.1016/j.cpnec.2026.100385.
