# Supplementary material for: Emotional dynamics during glucocorticoid treatment in chronic cluster headache – Insights from dynamic time warp analysis
Source: Compr Psychoneuroendocrinol. 2026 Oct 2;28:100385. doi: 10.1016/j.cpnec.2026.100385 (PMC13659764; doi:10.1016/j.cpnec.2026.100385)
Supplement: Supplementary file 1 [file mmc1.pdf]

Appendix A – Ecological momentary assessment (EMA) questionnaire

Item		Question	Answer
1	Feeling good	How do you feel at this moment?*	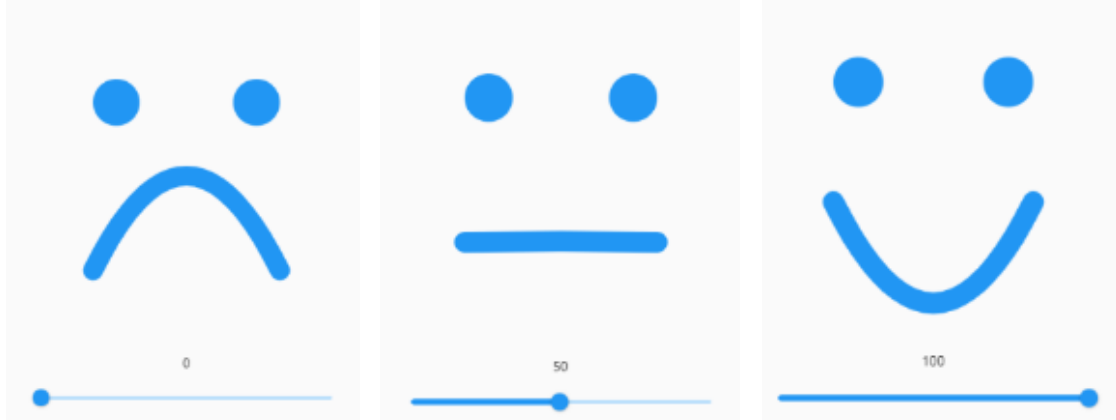
2	Happy	I am …	Very unhappy (0) – Very happy (100)
3	Sleeping problems**	Last night I slept …	Very bad (0) – Very well (100)
4	Tense**	I feel …	Very tense (0) – Very relaxed (100)
5	Upset	I feel …	Not upset (0) – Very upset (100)
6	Cheerful	I feel …	Not cheerful (100) – Very cheerful (100)
7	Irritation	I feel …	Not irritated (0) – Very irritated (100)
8	Motivation	I feel …	Very unmotivated (0) – Very motivated (100)
9	Sad	How sad do you feel at this moment?*	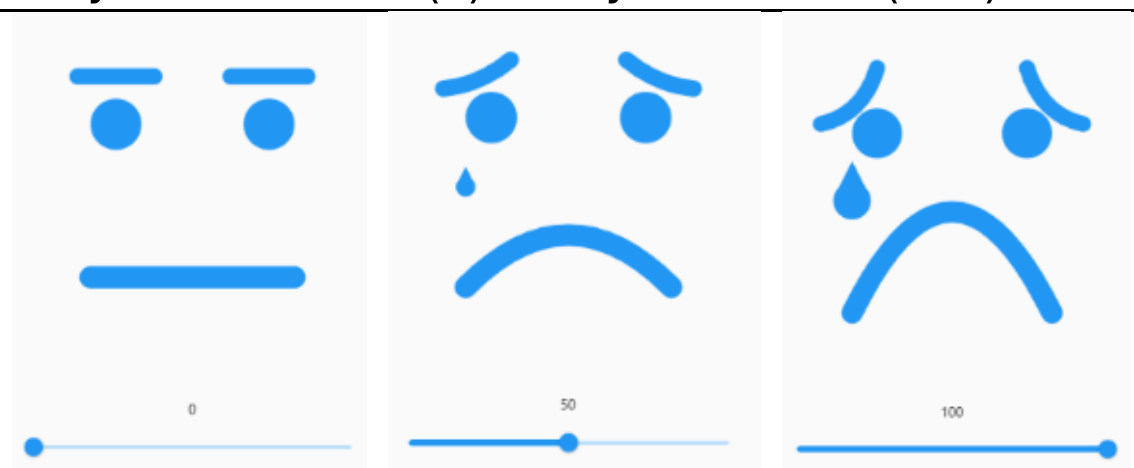
10	Energetic	I feel …	Not energetic (0) – Very energetic (100)
11	Enthusiasm	I feel …	Not enthusiastic (0) – Very enthusiastic (100)
12	Nervous	I feel …	Not nervous (0) – Very nervous (100)
13	Bored	I feel …	Not bored (0) – Very bored (100)
14	Calm	I feel …	Not calm (0) – Very calm (100)
15	Anxious	How anxious do you feel at this moment?*	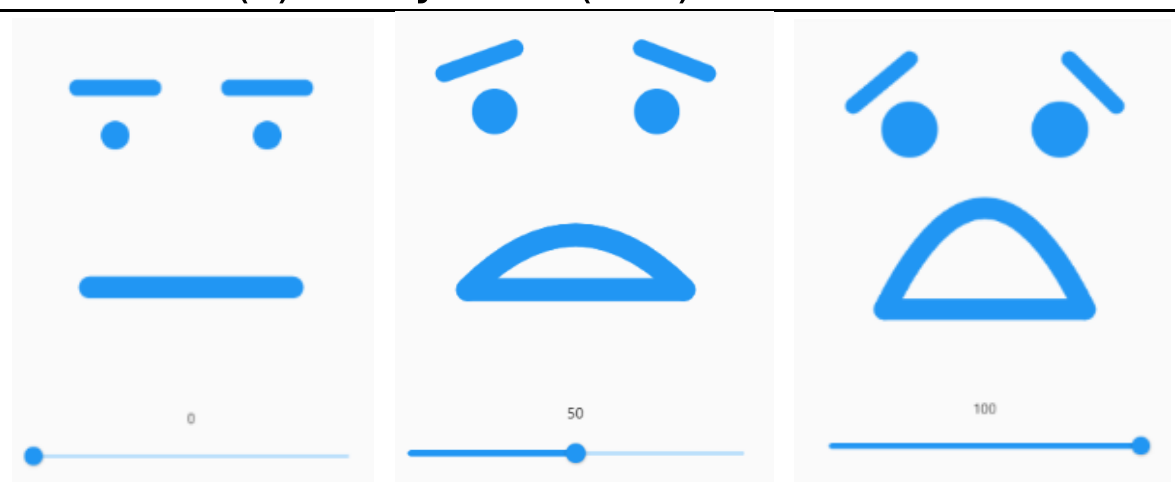
16	Guilty	I feel …	Not guilty (0) – Very guilty (100)
17	Concentration	I can …	Concentrate very poorly (0) – Concentrate very well (100)
18	Hopeful	I feel …	Very hopeless (0) – Very hopeful (100)
19	Tired	I feel …	Not tired (0) – Very tired (100)
20	Physical discomfort	I'm experiencing	No physical discomfort (0) – Very much physical discomfort (100)
21	Worrying	I …	Do not worry (0) – Worry a lot (100)
22	CH bother**	To what extent are you bothered by your cluster headache at this moment?*	A lot (0) – Not at all (100)
23	Attack frequency	How many cluster headache attacks do you have at this moment?*	1. No attacks<br>2. 1 attack<br>3. 2-4 attacks<br>4. 5-10 attacks<br>5. >10 attacks

All questions were answered related to the past 24 hours. Questions 1-22 were answered with sliders ranging from 0 to 100; question 23 was multiple choice. *It was

specified to participants that ‘at this moment’ indicated the current questionnaire and thus the past 24 hours. ** The scores of items 3, 4 and 22 were reversed in the analysis to ensure consistency across all items, such that a score of 0 represents 'not experiencing the item at all' and 100 represents 'experiencing the item very much’.
