# Supplementary material for: Emotional dynamics during glucocorticoid treatment in chronic cluster headache – Insights from dynamic time warp analysis
Source: Compr Psychoneuroendocrinol. 2026 Oct 2;28:100385. doi: 10.1016/j.cpnec.2026.100385 (PMC13659764; doi:10.1016/j.cpnec.2026.100385)
Supplement: Supplementary file 2 [file mmc2.pdf]

Appendix B – Individual (idiographic) analyses

Panel A (left): CH symptoms; portrays standardized scores of CH burden and attack frequency. A high score indicates no treatment response and a low score indicates good treatment response.

Panel B (middle): undirected network and for all ten participants.*

Panel C (right): directed network for all ten participants.*

* The size of each node (panel B + C) is proportional to its connectivity. The darkness and thickness of the lines (undirected) and arrows (directed) between the nodes correspond to the strength of the effect, with darker colors and bolder lines/arrows indicating stronger effects. Green and red lines/arrows represent positive and negative effects respectively.

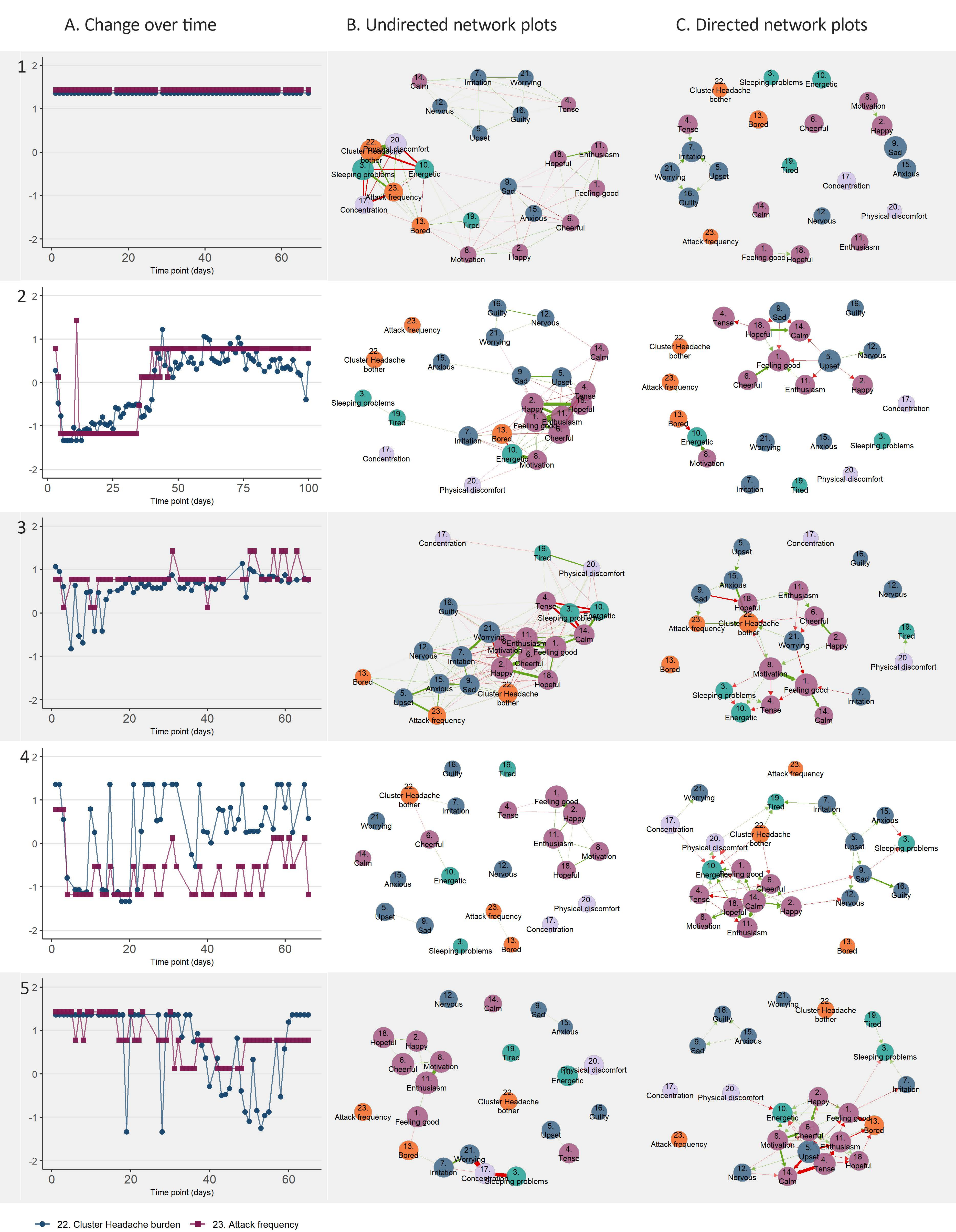

A. Change over time
B. Undirected network plots
C. Directed network plots
1
2
3
4
5
Time point (days)
22. Cluster Headache burden
23. Attack frequency
1. Feeling good
2. Happy
3. Sleeping problems
4. Tense
5. Upset
6. Cheerful
7. Irritation
8. Motivation
9. Sad
10. Energetic
11. Enthusiasm
12. Nervous
13. Bored
14. Calm
15. Anxious
16. Guilty
17. Concentration
18. Hopeful
19. Tired
20. Physical discomfort
21. Worrying
22. Cluster Headache bother
23. Attack frequency

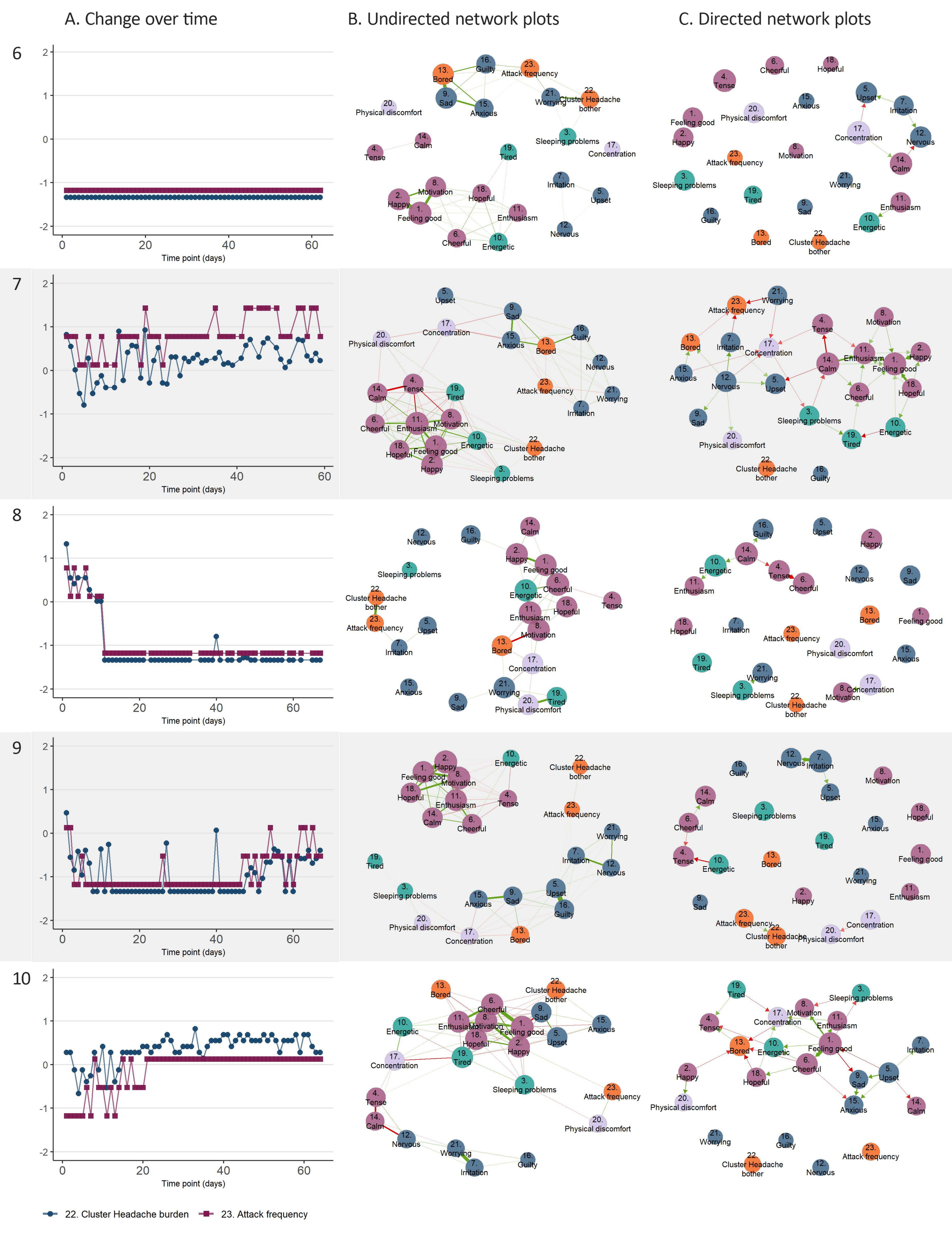
A. Change over time
B. Undirected network plots
C. Directed network plots
6
7
8
9
10
Time point (days)
22. Cluster Headache burden
23. Attack frequency
