# Supplementary material for: Emotional dynamics during glucocorticoid treatment in chronic cluster headache – Insights from dynamic time warp analysis
Source: Compr Psychoneuroendocrinol. 2026 Oct 2;28:100385. doi: 10.1016/j.cpnec.2026.100385 (PMC13659764; doi:10.1016/j.cpnec.2026.100385)
Supplement: Supplementary file 3 [file mmc3.docx]

**Plain language summary**

Cluster headache is sometimes treated with prednisone, but both cluster headache and prednisone can affect mental health. We studied the relation between emotions, prednisone and cluster headache, and found that mental health worsened during prednisone use, regardless of cluster headache symptoms, and emotions have a stronger relation with how much patients are bothered by their cluster headache than the number of attacks. Therefore, physicians should assess mental wellbeing before and during prednisone use, so they can give personalized treatment advice.

**Plain language summary publication**

**Main Finding:** in our headache clinic, we observed negative changes in mental wellbeing during prednisone treatment in people who used prednisone for cluster headache, even if cluster headache symptoms improved during treatment. Additionally, we found that how much a person is bothered by their cluster headache is more closely related to emotions than the number of attacks.

**Motivation (Problem):** Cluster headache can have a negative impact on mental health due to the severe attacks. For example, people with cluster headache are more likely to experience depression or anxiety. When other cluster headache medication is not effective, a short course of prednisone can be used. However, we know that prednisone can have a negative impact on mental health as well.

**Methods:**

In this study, people with chronic cluster headache completed a short, daily questionnaire during treatment with prednisone. The questionnaire monitored emotional wellbeing, physical wellbeing and cluster headache symptoms. Because the questionnaire was filled out every day during treatment, we could check if certain symptoms came before or after others.

**Results:** We found that positive emotions decreased and negative emotions increased during treatment with prednisone. These changes were found in all participants, regardless of whether their cluster headache improved, worsened or stayed the same. Feelings of upset, hopelessness and low motivation were most likely to be followed by changes in other emotions. The number of cluster headache attacks per day was not related to how much people felt bothered by their cluster headache. However, changes in emotions were related to how much people were bothered by their cluster headache.

**Implications:** We showed that there were negative emotional changes during prednisone treatment. Therefore, these potential side effect should be discussed with patients before starting with prednisone treatment. In the future, digital tools might help monitor emotions during prednisone treatment.
